# Assessment of the knowledge of the modes of transmission and prevention of malaria among pregnant women attending antenatal clinic at the Nkwen Health Center Bamenda, Cameroon

**DOI:** 10.11604/pamj.2019.33.137.16896

**Published:** 2019-06-24

**Authors:** Claude Ngwayu Nkfusai, Samuel Nambile Cumber, Fala Bede, Tabe Armstrong Tambe, Joyce Mahlako Tsoka-Gwegweni

**Affiliations:** 1 Department of Microbiology and Parasitology, Faculty of Science, University of Buea, Buea, Cameroon; 2 Cameroon Baptist Convention Health Services, Yaoundé-Cameroon; 3 Section for Epidemiology and Social Medicine, Department of Public Health, Institute of Medicine (EPSO), The Sahlgrenska Academy at University of Gothenburg, Box 414, SE-405 Gothenburg, Sweden; 4 Faculty of Health Sciences, University of the Free State, Bloemfontein, South Africa; 5 School of Health Systems and Public Health, Faculty of Health Sciences, University of Pretoria Private Bag X323, Gezina, Pretoria, South Africa; 6 Department of Nursing, Institute of Health and Biomedical Science, Cameroon Christian University Bali, Bali, Cameroon

**Keywords:** Knowledge, mode of transmission, malaria, pregnant women, antenatal clinic, Nkwen Health Centre

## Abstract

**Introduction:**

Malaria is a life threatening disease caused by the Plasmodium parasite, transmitted through the bites of infected female anopheles' mosquitoes. According to the latest WHO data published in 2017, malaria deaths in Cameroon reached 9.161 deaths accounting for 4.14% of total deaths. The age adjusted death rate is 29.11 per 100,000 and Cameroon is ranked the 30th in the world with a high prevalence of malaria. The aim of this study was therefore, to access the knowledge of the modes of transmission and prevention of malaria among pregnant women attending Antenatal Clinic (ANC) at the Nkwen Health Center, Bamenda.

**Methods:**

This was a cross-sectional hospital based survey study. The researchers recruited 51 eligible women in the Nkwen Health Centre and used a validated and pre-tested questionnaires to collect data. Collected data were entered into Excel and analysed using descriptive statistics and the results presented in tables and figures.

**Results:**

Sixty four percent of the women have basic knowledge about the mode of malaria transmission. Thirty six percent of the women had little knowledge about malaria transmission modes and the possible dangers of the disease.

**Conclusion:**

Slightly above 50% of pregnant women have basic knowledge on the modes of malaria transmission. Lack of knowledge regarding the modes of malaria transmission can be one of the reasons why there is still quite a high level of malaria prevalence among pregnant women attending ANC at the Nkwen Health Center, Bamenda. There is therefore, a need to educate women on malaria transmission modes.

## Introduction

Malaria is a mosquito-borne infectious disease affecting humans and other animals caused by parasitic protozoans (a group of single-celled microorganisms) belonging to the plasmodium type [1]. Malaria is caused by four different species of the protozoa of the genus *Plasmodium* which can be transmitted by the bite of a female anopheles mosquito, congenitally or through exposure of infected blood products [2]. According to studies by Miller and colleagues, pregnant women are more likely than the non-pregnant women to become infected with malaria and have severe infection [3]. The dangers of malaria in pregnancy includes but are not limited to, preterm delivery, low birth weight, spontaneous abortion, stillbirth congenital infection, and maternal death [3]. Malaria is the second most common cause of infectious disease related death in the world after tuberculosis. Malaria is estimated to affect 350 to 500 million people annually and accounts for 1 to 3 million deaths per annum. According to WHO Fact Files, nearly half of the world's population is at risk of contracting malaria. Increased prevention and control have led to a 29% reduction in malaria mortality rate. More so, Sub-Saharan Africa has the highest share of the malaria burden [4].

In Cameroon, malaria is the leading cause of hospital visits and deaths with children and pregnant women being the most affected. Everyone in Cameroon is considered at high risk of contracting malaria [5]. According to the Permanent Secretary of the National Malaria Control Program (NMCP), at least 2 million cases of patients suffering from malaria are recorded in health facilities each year and at least 4,000 people dying from the disease. This accounts for a 33.3% prevalence rate of morbidity and 30% mortality rate [4, 5]. In Cameroon, measures such as proper hygiene drainage have been put in place to avoid stagnant waters which serve as mosquitoes breeding grounds, distribution of free Long-lasting Treated Insecticides Mosquito Bed nets (LITNs) at ANC clinics for pregnant women, as well as doing door-to-door distribution of bed nets [6]. Scaling up of affordable anti-malaria treatment using combination of anti-malaria drugs as part of the push to end malaria, whereby patients pay less than 1,000 FCFA for several days treatment which has also been one of the approaches to curbing the disease. Moreover, other measures such as free intermittent treatment for pregnant women as from four months of pregnancy, free treatment for children less than five years old have been of great help. However, health officials say universal protection and effective treatment are paying off [7]. The aim of this study is therefore, to access the knowledge of the modes of transmitting and preventing malaria among pregnant women attending Antenatal Clinic in the Nkwen Health Center, Bamenda.

## Methods

**Study design, study area and population:** this study was a cross-sectional hospital-based survey carried out at the PMI Medicalized Health Center Nkwen in Mile 2 Bamenda which is found in the Northwest Region of Cameroon. The PMI Medicalized health center consists the following unit: emergency units, gynecological unit, medical and surgical unit, diabetic unit, and ANC and postnatal unit. This study was carried out specifically at the ANC clinic during the visiting hours.

**Inclusion criterion:** this study involved all pregnant women who came for ANC at the Nkwen Health Centre, Bamenda during the study period (1-21 March 2018) and who gave their consent either in written or verbal forms.

**Exclusion criterion:** the study excluded pregnant women who came for ANC but never gave their consent to participate.

**Sample size and Sampling:** the sample size was exhaustive. A total of 78 pregnant women came for ANC during the study period (1-21 March 2018). However, out of these number, only 51 pregnant women gave their consent to take part in the study and were administered questionnaires.

**Administration questionnaires:** questionnaires were administered among consented participants in the health center who agreed to participate in the study. The questionnaires captured data on socio-demographic characteristics, and level of the women's awareness on the malaria transmission modes.

**Data collection:** this was a health center based survey where all participants who consented were interviewed using a structured questionnaire filled at the health center. Prior to the use of the questionnaires in the study by participants, a total of 5 questionnaires were pretested among pregnant women attending ANC at the Saint Louis Clinic in Bamenda with the aim of revising poorly structured questions, estimating the average time required to fill the questionnaire and by so doing validating the use of the questionnaire in study. It was estimated that, each questionnaire could be administered for 20-30 minutes after the pretest. A total of 51 questionnaires were administered to the pregnant women attending ANC at the the Nkwen Health Centre for a period of 3 weeks to assess their level of awareness and knowledge on malaria transmission modes. Knowledge on malaria transmission modes was tested through 8 questions. Each correct response was scored as 1 and 0 for a wrong response. The knowledge scores for an individual was calculated and summed up to give a total knowledge score on 8. A score between 0-3 was classified as poor, 4-6 as good and 6-8 as excellent for knowledge malaria transmission modes. This was adapted from a study conducted by Mazigo HD *et al.* [8].

**Data analyses:** data were entered into Excel. The data were verified for completion and incomplete entries were deleted, cleaned and analysed. Descriptive analysis was carried out by calculating the mean and frequencies of different variables. Results were presented in the form of frequency tables and charts

**Ethical and administrative consideration:** ethical clearance was obtained from the Institutional Review Board of the Cameroon Baptist Convention Health Services. Administrative clearance was obtained from the Regional Delegation of Public Health for North West Region Cameroon. Participants had the study protocol carefully explained to them and participation was voluntary. Written and verbal informed consent was obtained from all participants.

## Results

**Socio-demography characteristics of respondents:** the majority of the women (88%) were between the ages 15-30 years (adolescents and young women), while 12% of the women fell between the ages 36-40 years old. The study also revealed that a greater percentage (64.7%) of the women were not married compared to others. The table also reveals that 58.2% of the women were within the 3rd trimester compared to the others who were within the 1st and 2nd trimester of their pregnancies. We can also deduce from the table that 29% of the women have had more than one pregnancy compared to the other 41.7% who were pregnant for their first time (Table 1).

**Table 1:** Socio-demography characteristics of respondents

Variable		Frequency /number	Percentage (%)
Age Range ( years)	15-20	15	29.4
	21-25	18	35.2
	26-30	12	23.4
	31-35	3	5.88
	36-40	3	5.88
	**Total**	51	100
Marital Statues	Married	33	64.7
	Single	6	11.7
	Separated	6	11.7
	Divorce	3	5.5
	Widow	3	5.5
	**Total**	51	100
Duration of pregnancy (Month)	0-3	6	11.7
	4-6	15	39.4
	7-9	30	58.2
	**Total**	51	100
Number of Pregnancies	1^st^ pregnancy	24	47.1
	2^nd^ pregnancy	12	23
	More than 2	15	29
	**Total**	51	100

**Figure 1:**
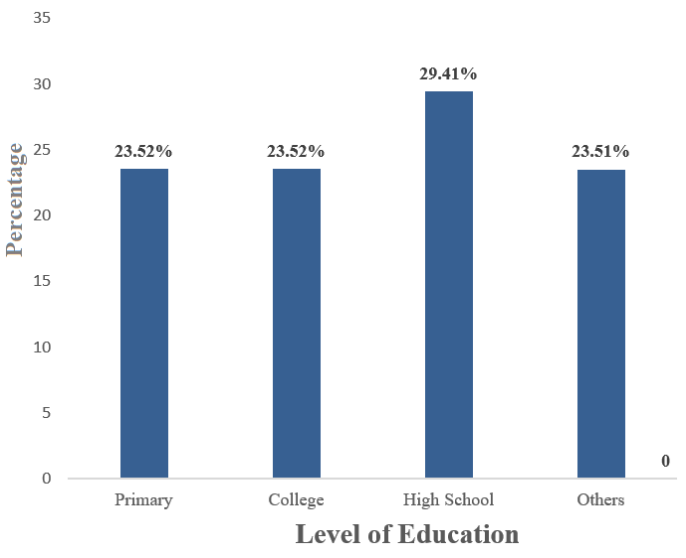
Distribution of respondents by level of education

**Distribution of respondents by level of education:** there was a greater percentage (52.92%) of the women who had had at least high school level of education as compared to the others (47.08%) who hadn't attained a high school level of education (Figure 1).

**Figure 2:**
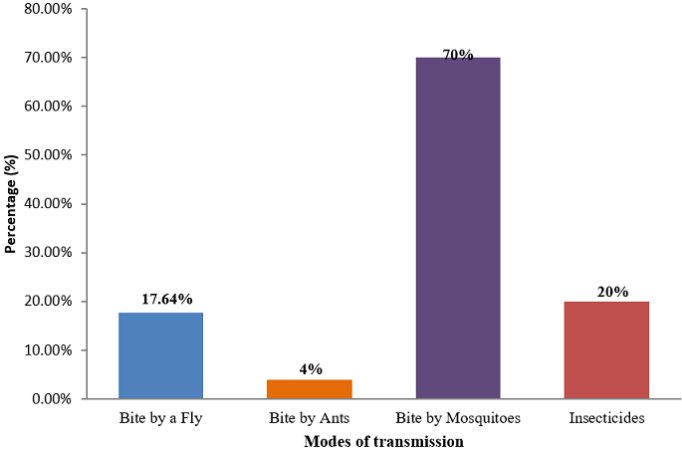
Knowledge on malaria transmission modes

**Knowledge on malaria transmission modes:** it was seen that, 70% of the women know that mosquitoe bites are the principal malaria transmission modes (Figure 2).

**Figure 3:**
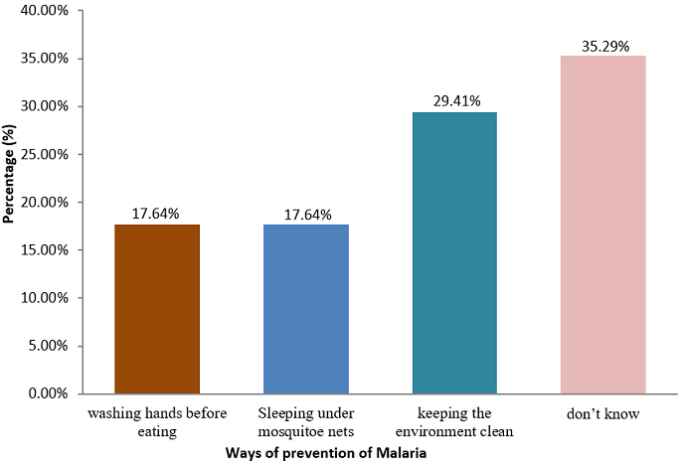
Ways of preventing malaria

**Ways of preventing malaria:** about 46% of the women know that sleeping under treated mosquito nets and keeping our environment clean were the effective ways of malaria prevention compared to a majority who still didn't have effective knowledge on the prevention of malaria (Figure 3).

**Occurrence of malaria during pregnancy:** a greater percentage of the women (70.8%) have experience malaria at least once during their pregnancy as compared to a few (29.1%) who hadn't had malaria during their pregnancy (Table 2).

**Table 2:** Occurrence of malaria during pregnancy

Occurrence	Frequency	Percentage (%)
Have not had malaria during my pregnancy	15	29.1
Have had malaria once	21	41.7
Have had malaria once twice	15	29.1

**Knowledge of dangers of malaria:** at least 52% of the women are aware of the dangers of malaria while 76.4% of the women knew malaria causes death. This demonstrates that a majority of the women are aware of the dangers of malaria (Table 3).

**Table 3:** Knowledge of dangers of malaria

Variables	Response	Frequency	Percentage
Do you know the dangers of malaria?	Yes	27	52.9
	No	24	47.1
	**Total**	51	100
Do you know malaria is kills?	**Response**	**Frequency**	**Percentage**
	Yes	39	76.4
	No	6	11.7
	Don’t think so	6	11.7
	**Total**	51	100

## Discussion

This study was conducted to access pregnant women's knowledge on malaria and the various transmission modes at the Nkwen CMA Health Center Bamenda. According to the data collected, 64% of the women had a good knowledge of malaria as compared to the 36% who had limited knowledge on the disease. This is in line with another study carried out in Tanzania which states that a reasonable 56% of the women were aware of the modes of transmission, prevention and treatment of malaria [8].

This study also shows that a majority (70%) of the pregnant women know exactly the main mode of malaria transmission which is through mosquito bites. This also supports the findings of a similar study carried out in Buea Health District, Cameroon by Helen *et al.* [9] which stated that 86% of pregnant women and their care takers were aware of the correct malaria transmission modes. However, this sharply contrasts with a new study in Ndu, north west region Cameroon [10] 9 years ago which stated that just 27.9% of the population where aware of the major malaria transmission modes. This shows that knowledge on malaria was strongly associated with level of formal education as can be explained by the 52.9% of those who have attained at least high school education and have been taught lessons on malaria in schools and are also more liable to read, listen and comprehend malaria messages. Thus, education remains a powerful tool to empower people to make positive decisions for themselves and their families.

The study also display that 70.8% of the pregnant women have had malaria at least once during their pregnancy which ties in with the fact that pregnant women are 3 times more likely to suffer from malaria infection and have a higher mortality rate as compared to their non-pregnant counterpart [11]. Also a majority of the women knew about malaria and the risk it poses which is also in line with a similar study by Helen *et al.*[9] in Cameroon on pregnant women and care givers knowledge on the effects of malaria in pregnant women. A majority (66.4%) knew at least one or more side effects of malaria in pregnant women. They were able to give effects but not limited to anemia, abortion and fetal and maternal deaths.

**Limitation of the study:** the researchers faced some challenges during this research; the study had a small sample size and was a facility-based research. The limited scope didnt give room for extensive research and pronuncement. Furthermore, studying self-reported knowledge is itself a limitation because one cannot rely completely on the information provided by the participants because of recall and social desirability biases. Despite these shortcomings, this study provides relevant information in the context of malaria in pregnancy in the Nkwen Health Center, Bamenda.

## Conclusion

Despite concerted efforts and measures taken such as continuous education on malaria, distribution of LITNs and subsidized malaria treatment just to name a few, malaria continues to be one of the killing diseases even among pregnant women in the Nkwen neighborhood. There is need for continues sensitization as this will go a long way to reduce its prevalence since many people will be educated and will be more aware of their environment. This paper reveals that slightly above 50% of pregnant women have basic knowledge on the modes of malaria transmission. There is lack knowledge regarding malaria transmission modes which accounts for the continues high prevalence rate of malaria among pregnant women attending ANC at the Nkwen Health Center, Bamenda. There is therefore, a need to educate women on malaria transmission modes. This paper recommends further studies on malaria as it still remains the number one killer disease that claims more life in Cameroon and the world over. Also, these researchers challenge the state of Cameroon to embrace and take the education of the public on malaria prevention very serious. The government should adopt policies that will ensure clean environments to curb the spread of the diseases in the country.

### What is known about this topic

- Malaria is more prevalent among pregnant women than their non-pregnant counterpart as a result of their weaken immune system;
- Malaria still remains the number one leading cause of death among pregnant women and children in the world;
- The dangers posed by malaria during pregnancy are not limited to the mother but have an adverse effects on the unborn baby which cannot by any means be taken for granted.

### What this study adds

- The study reveals that slightly above 50% of pregnant women attending ANC at the Nkwen Health Center, Bamenda had basic knowledge on malaria. There is need for admonishment of the women on the dangers of malaria and how to avoid being infected;
- This study will serve as a reference document for government, policy makers and stakeholders in decision making;
- The article lays a path to guide them in the implementation of projects regarding the curbing of malaria in the country and region as a whole.

## Competing interests

The authors declare no competing interests.
